# [Retracted] Downregulated microRNA-200a promotes EMT and tumor growth through the Wnt/β-catenin pathway by targeting the E-cadherin repressors ZEB1/ZEB2 in gastric adenocarcinoma

**DOI:** 10.3892/or.2026.9045

**Published:** 2026-01-12

**Authors:** Ningning Cong, Ping Du, Anling Zhang, Fajuan Shen, Juan Su, Peiyu Pu, Tao Wang, Jie Zjang, Chunsheng Kang, Qingyu Zhang

Oncol Rep 29: 1579–1587, 2013; DOI: 10.3892/or.2013.2267

Subsequently to the publication of the above paper, a concerned reader has drawn to the Editor's attention that, for the immunohistochemical data shown in Fig. 2E, the same data panel had apparently been included for the ‘ZEB1/Con’ and ‘SEB2/Min’ experiments. In addition, for the Snail2 experiments shown in Fig. 3A, the Snail Con(trol) and Snail Mimi panels looked strikingly similar, even though the intensity of the antibody (red) channel appeared to have been decreased in the Mimi panel. Finally, for the immunohistochemical images shown in Fig. 3C, the E-cadherin Con(trol) and Scr panels appeared to show a region of overlap, suggesting that these data were derived from the same original source, where the results of differently performed experiments were intended to have been portrayed.

Given that it has come to light that this trio of figures had apparently been assembled incorrectly, which might have had an adverse effect on the interpretation of the results and conclusions in the article, the Editor of *Oncology Reports* has decided that this paper should be retracted from the Journal on account of a lack of confidence in the presented data. The authors were asked for an explanation to account for these concerns, but the Editorial Office did not receive a reply. The Editor apologizes to the readership for any inconvenience caused.

